# COVID-19 and its impact in the dental setting: A scoping review

**DOI:** 10.1371/journal.pone.0244352

**Published:** 2020-12-18

**Authors:** Bashier Ahmed Kathree, Saadika B. Khan, Rukshana Ahmed, Ronel Maart, Nazreen Layloo, Winifred Asia-Michaels

**Affiliations:** Department of Restorative Dentistry, Faculty of Dentistry, University of the Western Cape, Cape Town, South Africa; Universidad de Antioquia, COLOMBIA

## Abstract

**Introduction:**

The scoping review examined the evidence related to infection control and transmission measures of the SARS-CoV-2 virus in a dental setting during this pandemic. Dental practitioners are normally guided in practice by set ethical principles, thus the researchers wanted to determine how these rules are managed during this pandemic.

**Methods:**

A protocol specific for the objectives of this study was developed according to the criteria for a scoping review. Relevant databases (Pubmed, Scopus, Elsevier, Science Direct, Wiley), including online access to health/ dental organizations (World Health Organization/ American Dental Association), were searched to identify evidence which was restricted to the English language for the period 2015–2020. Predetermined eligibility criteria were applied, evidence was assessed and data extracted for each included article. Relevant outcomes assessed were: infection control measures, transmission of the SARS-CoV-2 virus, such as modes and sources of transmission and the ethical principles related to the dental setting with a focus on the COVID-19 pandemic.

**Results:**

Searches yielded a total of 402 articles: 387 from electronic databases and 15 from other sources. Of these, 231 were unrelated to the objectives of the current scoping review. The full text of 69 studies was assessed for eligibility, of which 26 were finalized for inclusion following the objectives and inclusion criteria set for the scoping review. Most of the included articles were reviews, recommendations and guidelines for dentists. A narrative explanation of the pre-specified outcomes is reported for the 3 areas covered for this review. There is no clinical evidence available that can support the recommendations by individuals, dental organizations or health authorities related to the objectives of this review, but these may be considered as the much needed guidelines during the unprecedented COVID-19 pandemic. A different ethical framework is required during a pandemic and these must be informed by evidence.

## Introduction

Corona virus disease (COVID-19) which started as an epidemic in Wuhan, China was subsequently declared a pandemic by the World Health Organization (WHO) as it has wreaked havoc across different nations and continents for months now [1]. It has affected many lives, people from different socio-economic backgrounds and cultures and professional backgrounds, but especially all healthcare professionals who are in the front line of combating the disease [1–7]. Dental professionals are particularly at risk due to the nature of their clinical work. Well-meaning dentists who want to serve the needs of their patients as they should may be spreading the virus faster and extensively, without being aware of it [2, 5, 7]. Therefore guidelines and standard operating procedures, which encompass their clinical work, work space as well as the principles guiding their practices, must be well formulated and strictly followed to protect themselves, dental staff and the patients they serve [2–9]. But what is it about the nature of their clinical work that places them and their patients at greater risk of developing COVID-19 or any similarly infectious viral diseases? And, having committed to the principles of the Hippocratic Oath, are dental practitioners able to evade these ethical principles that guide their practices? The focus of this scoping review is therefore to recognize how the nature of dental work may contribute to the spread of COVID-19 and how this may be circumvented whilst remaining committed to set ethical guidelines.

With current world news emphasizing the strict personal hygiene control measures required for combating COVID-19 and its transmission, people all over the world have become even more conscientized when visiting health centres, including dental clinics, be it for any kind of treatment (restorative, prosthodontic or dental extractions). The number of patients attending dental clinics at the start of the pandemic was substantially reduced globally, for fear of contracting the COVID-19 infection when exposed to the environment and/ or other humans. Moreover, country specific health authorities and dental organizations have at such an unprecedented time realized the importance of their roles related to developing and modifying practice protocols and guidelines as required and how these changes need to be emphasized to guide dental practitioners [2–5].

People of all ages are at risk of contracting COVID-19, but the vulnerable elderly, those who are immunocompromised, those who have severe respiratory conditions or other comorbidities are particularly susceptible [1–7]. Similarly, those who are in contact with infected and suspected patients, such as your healthcare workers and other patients present in a clinic are at a greater risk of developing the infection. There is currently no evidence from quality research for approved treatment, vaccines, cures or other biomedical prevention options for COVID-19 or patients diagnosed positively with SARS-CoV-2 [1–7]. Therefore, the focus must be on controlling the source of the infection, preventing the development of infection amongst the population and on prioritizing measures to lower the risk of transmission of COVID-19 [2–5].

The SARS-CoV-2 virus also remains on surfaces for extended periods of times (hours to days) depending on environmental temperatures, humidity and the type of surface material [1]. Thus, utilizing proper and effective disinfection control measures within dental settings cannot be over emphasized. In addition, the dental personnel must wear personal protective equipment (PPE), to prevent being infected by symptomatic and asymptomatic patients [2]. Additionally, also new surfaces with anti-infective nano-coatings have been proposed for health settings [8].

During a pandemic, there will be times when dentists will have no choice but to treat patients presenting with emergency procedures, such as pain, bleeding and sepsis [6, 7]. What is important is that basic infection control measures including special disinfection and transmission-based precautions (TBP) will have to be utilized and strictly adhered to [9–11]. Dentists will have to insist on the practice of standard infection control or precautions (ICP) and the transmission-based precautions amongst their patients and particularly the practice staff [9–11]. The second-tier precautions which include contact, droplet and airborne types should especially be practiced in the dental setting to prohibit dentists, patients or other dental staff from becoming infected during delivery of dental procedures as these regularly produce aerosols and droplets which may carry the SARS-CoV-2 virus [9–11]. Thus, the risk of cross infection between dentist, dental personnel and patients are great [12].

Therefore, at the outset of the pandemic, the American Dental Association (ADA) called on dentists globally to postpone non-urgent dental procedures or those deemed not an emergency [2]. Similarly, the department of health and dental organizations of affected countries had announced similar protocols for the actions of dentists, all already deviating from normal ethical practices [13]. They were therefore encouraged to only treat pain and sepsis and using the strictest infection and transmission control measures. These guidelines were based on available communications, newly created practices and research from severely affected countries related to the routes of transmission, treatment measures with positive outcomes, and rising fatality rates [1–5]. Other types of dental treatment that need consideration during the pandemic, irrespective of aerosol production, include bleeding following dental procedures, oral cancers and fractures; these patients should be treated in appropriately set-up and ventilated rooms, especially if they tested positive for COVID-19 [6].

As dental practitioners, we are required to uphold all ethical principles, especially those of respect for autonomy, beneficence, non-maleficence, veracity and justice [14]. Whilst being cognizant of these principles, dentists are bound to follow guidelines set by their respective country’s health councils and public health authorities during a pandemic.

The rationale for this review is therefore to obtain the evidence related to infection control, transmission of SARS-CoV-2 or COVID-19 and the related ethical principles as dental educationalists at dental institutions need to guide others during a pandemic.

The aim of the study is to conduct a scoping review to identify the research that focus on infection control measures and transmission of SARS-CoV-2 or COVID-19 infections within a framework of accepted ethical principles.

The objectives of the review include:

1. To evaluate the literature focusing on ethics as it relates to the dental professional during the COVID-19 period;
2. To gauge the literature regarding infection control and disinfection measures, and transmission of the SARS-CoV-2 during the pandemic and
3. To identify evidence that may serve to guide practitioners regarding these infection control measures and transmission of COVID-19 in a dental setting.

*The research question/s for the scoping review state/s*:

What infection control and transmission measures can be identified that may inhibit spread of SARS-CoV-2 or COVID-19 in a dental setting?

Can ethical principles set for normal clinical dental practice adequately address concerns during a pandemic?

## Methodology

A study protocol was developed to guide researchers for this scoping review, but it was not published. A group of researchers looked at the evidence on COVID-19 related to the dental profession with a special focus on infection control, transmission of infections, the ethical principles related to these and challenges it presents in a dental clinical setting [15, 16]. The design of the scoping review framework is based on the Joanna Briggs Institute (JBI) Framework of evidence synthesis which consist of 5 stages as illustrated in Table 1 [16].

**Table 1:** Joanna Briggs Institute framework of evidence for scoping reviews.

PROCESS	DETAILS
1. Identifying the research question	Clarifying and linking the purpose and research question
2. Identifying relevant studies	Using the three-step literature search in order to balance feasibility with breadth and comprehensiveness
3. Study selection	Careful selection of the studies using a team approach and including all levels of evidence considered by the JBI levels of evidence
4. Presenting the data	Charting the data in a tabular and narrative format where applicable
5. Collating the results	Identifying the implications of the study findings for policy, practice or research

After specifying the research question, the focus of the inclusion criteria related to the nature of published SARS-CoV-2 or COVID-19 information, with a special focus on the dental profession. The criteria thus searched for, related to infection control and disinfection measures, transmission of SARS-CoV-2 infections and the set ethical principles associated with treating dental patients and managing dental staff.

The outcomes for the review include *infection control measures*, *transmission of infections* (mode, source and prevention) and the *associated ethical practices* and the impact on the dental setting during this period of COVID-19. Recommendations, standard operating procedures and guidelines for the COVID-19 pandemic were reported as well. The literature was limited to the English language and included evidence from different health settings (dental, hospital and clinical practice), different study designs but was not limited to primary research; thus, included opinions, reviews, guidelines and recommendations from oral health groups and organizations for the period 2015/01/01-2020/05/30. Research related to other areas of dentistry not covered by this scoping review was excluded.

The following databases and scientific working group websites were searched for relevant publications: PubMed, Science Direct, Scopus, Elsevier and Wiley, and including the following health groups: WHO, Federation Dentaire Internationale (FDI) of the World Dental Federation, Centre for Disease Control (CDC), ADA; South African Dental Association (SADA) and Health Professions Council of SA (HPCSA).

The key terms and Medical Subject Headings were combined using Boolean operators; an example of one such a search string created and used includes:

(COVID-19 OR corona virus OR SARS-CoV-2) AND (dental clinics OR dental hospital OR dental institutions OR dental teaching clinic) AND (infection control OR disinfection) AND (literature reviews OR reviews OR observational OR clinical trials OR randomized controlled trials OR qualitative research OR systematic reviews) AND (2015/01/01-2020/05/30.

The staff in the Department of Prosthetic Dentistry was divided into pairs for the different areas set for the scoping review and to complete the searches from the different databases. The researchers excluded any duplicates selected from the different databases manually. They were also guided by study eligibility and data extraction forms created to select the appropriate articles and extract data independently (SK, BAK, RA, NL, RM, WA). As per three-step plan, articles were initially screened according to titles, abstracts of the selected titles reviewed next (BAK, RA, NL, RM, WA). Thereafter, data extraction was completed from the full text of included articles which was obtained from the different databases and the OH group websites (SK, BAK, RA, NL, RM, WA).

The different steps for the scoping review were all completed as per the inclusion and exclusion criteria and independently by the reviewers. For all the stages of the review, the two reviewers met and any discrepancies were resolved through discussion to determine final inclusion. Independent data extraction from the full text version of the selected studies was finalized between reviewers and consensus of data reached through discussion. When agreement could not be reached between the reviewers, a third reviewer adjudicated at all stages of titles and abstracts screening, and data extraction from full-text articles.

For analysis of the data, the search for articles is reported using a Preferred Reporting Items for Systematic reviews and Meta- Analysis (PRISMA) flow chart [17]. For all included studies, characteristics were analysed descriptively and followed the data extraction criteria, which needed to be modified slightly depending on the type of report e.g. when published, location, study design. A basic appraisal (i.e. value and relevance) of these included studies or articles was completed, though a specialized tool to evaluate the quality of the research reported as per the literature was not completed [15, 16]. The data are reported narratively using the criteria set for the objectives of the study.

## Results

### Finalization of searches (Fig 1)

**Fig 1:**
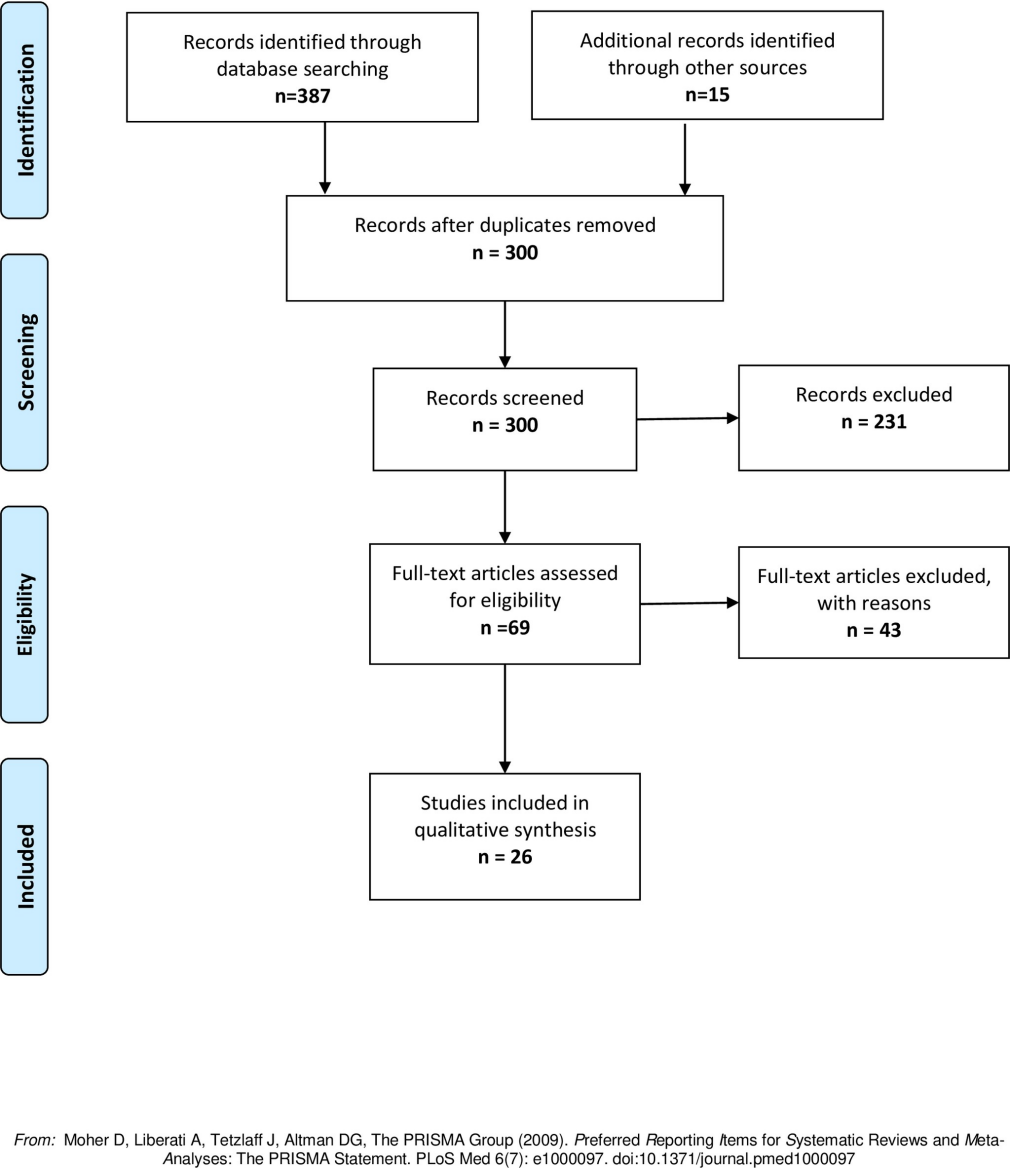
PRISMA flow diagram: Scoping review on impact of COVID-19 in a dental setting.

A total of 402 articles were retrieved, of which 102 were duplicates, and 231 were unrelated to the objectives of the current scoping review (Fig 1). After reading the abstracts, the full text of 69 studies were assessed for eligibility, of which 26 were finalized for inclusion following the objectives and set inclusion criteria (Fig 1). Fourteen of the included articles focused on *infection control measures* in the work place and how it relates to infrastructure, staff and patients (Table 2) [1, 6, 18–29]. In addition, other related aspects covered in these papers included clinical experiences, guidelines and recommendations for dental personnel, and attitudes and opinions of researchers [18, 19, 21, 23, 26–28]. Eight of the included articles focused on *transmission of infections* in a dental setting during a pandemic, pathway of spread and the different measures it could be conveyed from person-to-person (Table 3) [1, 6, 9, 21, 30–33]. The five *ethics articles* included for the review largely focused on the main principles of ethics (autonomy, beneficence, non-maleficence, veracity and justice), and associating these to the safety of individuals (patients and staff) and communities, the processes related to virus transmission and infection control measures (Table 4) [14, 34–37].

**Table 2:** Outcomes of infection control measures of included studies.

		Study Details	Study Outcomes	
	Titles	Author Country	Infection Control Measures	Guidelines or Recommendation for Dentists
1.	**Coronavirus Disease 2019 (COVID-19): Emerging and Future Challenges for Dental and Oral Medicine** J Dent Research 2020, Vol. 99(5) 481–487	L. Meng, F. Hua, Z. Bian China.	Staff: • Dental emergencies, • Hand hygiene, • PPE incl. gloves, gowns & goggles or face shields and N95 or FFP2 masks • Four-handed Dentistry beneficial for controlling infection, • Disinfect 2x daily • Disinfectants for touchable and surface tops Patients and accompanying people: • Wear masks, • Effective hand hygiene	• No consensus on provision of dental services • Pre-treatment triages • record temp of staff and patients • Use of intraoral antimicrobial mouth rinses before treatment • Minimize treatment procedures that produce droplets or aerosols and coughing such as the use of triple syringe and intra-oral radiographs. • Use of saliva ejectors. • Rooms be ventilated/ negatively pressured. *Emergency cases*: • Use extraoral radiography • Rubber dams, • high volume ejectors, • Face shields and goggles essential. • High/low speed drilling with water spray.
2.	**Assessing Knowledge, Attitudes and Practices of practitioners** **regarding the COVID-19 pandemic: A multinational study** *Dent Med Problems* 2020; 57(1):11–17	S Krishnappa Kamate,F, S Sharma India, Mauritius	Surveyed 860 dentists across 5 continents on dental practices during covid-19 pandemic: • 79% discussed Preventive measures against COVID-19 with the patients • 99% took preventive dental measures against COVID-19	• No preventive dental measures outlined. • Recommends patients with Covid-19 symptoms be screened, isolated and referred for treatment. • Dentists to follow CDC and WHO guidelines for all other patients
3.	**COVID-19 Transmission in Dental Practice: Brief Review of Preventive Measures in Italy.** J Dent Research 2020; 1–9	R. Izzetti, M. Nisi, M. Gabriele, F. Graziani Italy	Prophylactic measures (4 stages): 1.Triage 2. Patient entrance into surgery–use infra-red thermo-scanners and screen for risk factors, History of contact with positive patient, delay treatment by 14 days. 3. Dental treatment: Emergency treatment only. 4. Post treatment management: disposal of PPE and disinfection of area.	-1 min 0,2%-1% Povidone, 0,05%-0,1% Cetyl pyridinium chloride or 1% Hydrogen peroxide mouth rinse, • Hand washing 5 times (2xbefore and 3x after) using 60%-85% hydro-alcoholic solution for 60 sec, • PPE: gloves, masks, protective outer garments, safety goggles or face shields. • limit aerosol generating and cough inducing procedures. • Use Rubber-dam. • Disinfecting surfaces (door handles, etc.) using 0.1% Sodium Hypochlorite solution. • disinfect ventilation system. • keep windows open • keep environment dry, • waiting room protocol: social distancing, minimum objects, limit number of patients, nothing to be taken into surgery rooms, use disposable protection sheets over surfaces. • De-robe PPE in room, • dispose of PPE and disinfect area. After procedure wait for 5min air change,
4.	**Approaches to the management of patients in oral and maxillofacial** **surgery during COVID-19 pandemic** *J Cranio-Max Fac Surgery 48 (2020) 521e526*	Matthias Zimmermann, Emeka Nkenke, Austria	• Triage: prioritize patients (delay elective procedures, only emergency), avoid overlapping of work among specialities and between dentist and specialists. • Keep staff to the absolute minimum people in operating room, • Surgical team only to enter after intubation • Use Water proof gowns, • Operating room to be negatively pressured and well ventilated, • In-patient to be tested & to wear gown and FFP mask without valve. • 15 min air time wait before cleaning can begin.	No specific Guidelines available in literature for maxillofacial surgeons as to what to wear when operating in head and neck region. • Training of all staff on use of PPE. • Use of surgical masks, gloves and goggles for patient consultations. • Patient to rinse mouth with sodium hypochlorite or Povidone solution. • Aerosol generating procedure such as high- speed hand-piece, tracheotomy, irrigation, abscess drainage, needs special attention. • Use high level protection masks (N95, FFP3)
5	**Evolution of COVID-19 Guidelines for University of Washington Oral and Maxillofacial Surgery Patient Care** *J Oral Maxillofac Surg -*:*1–11*, *2020*	K Panesar, T Dodson, J Lynch, C Bryson-Cahn, L Chew, J Dillon, USA	The levels of PPE to be worn as determined by the American Society for Testing and Materials are: Level 1: Low barrier, for procedures with a low amount of fluid, blood, aerosol exposure or spray, Level 2: Moderate barrier, for procedures with a light to moderate amount of blood, fluid, aerosol or spray. Level 3: High barrier, for procedures with a moderate to high amount of blood, fluid, aerosols or spray. • These are influenced by: 1. Type of procedure- Emergency vs routine or Emergency treatment only. 2. Symptomatic status. 3. Test results	All patients to be tested for Covid-19 • Negative pts: use standard conventional surgical PPE (surgical mask, face shield or goggles, gowns, surgical caps and gloves) • Positive pts: use aerosolized transmissible disease (ATD) precaution, i.e. ‘airborne, respiratory and contact precautions, negative pressure room, powered air purifying respirators [PAPRs] or N95 respirators, eye shield or goggles, gown, gloves surgical cap, and a trained observer for donning and doffing’). N95 masks to be face-fitted. Fluid Resistance.
6.	**What dentists need to know about COVID-19** *https*:*//doi*.*org/10*.*1016/j*.*oraloncology*.*2020*.*104741*	Maryam Baghizadeh Fini Iran	• Hand hygiene using alcohol-based solution or hand-wash. • PPE includes surgical gloves, face shields, eye-wear, surgical masks (N95 or FFP2), • pre-procedure mouth rinse (uncertain of chlorhexidine) • extra-oral radiographs, • rubber dam, • limit aerosol producing procedure, • single use instruments, • surface disinfection using 0,5% hydrogen peroxide or0,1% sodium hypochlorite solution, • dry environment, • prompt disposal of waste	• Avoid touching unnecessary areas (dentures, bite blocks) • Disinfect work area every time, • avoid procedures causing coughing or gag reflexes • Clinical tips: • Do tooth extractions in a supine position • (prevents from operating in respiratory tract). • During denture construction: avoid touching items in dental workplace after contacting items with saliva contamination. • Prosthodontic material (e.g., RPD, impressions) to be disinfected by intermediate level disinfectant. • Salivary suction carefully done, • use topical anaesthesia at back of mouth during impression procedure to prevent gag reflex.
7.	**Overview of transnational recommendations for COVID-19 transmission control in dental care settings.** *Wiley Online Library*, *Oral Disease*, *10.1111/ODI.13431*	Mohammed Jamal et al UAE	• Screening protocols: • Online patient assessments or telecommunications • Waiting rooms rearranged: • Social distancing; frequent cleaning; • PPE; • Preoperative mouth rinse; • Rubber Dam; • Positive patient referred to airborne isolation rooms	• Emergency treatment only • Triage room for consultations • PPE Types: N95 or equivalent (especially in aerosol generating procedures), surgical mask, protective eye-wear, disposable working cap, appropriate gloves, gowns, impermeable shoe covers. • Avoid taking intra-oral radiograph. • Double barrier for intra-oral sensor or films, if intra-oral radiograph is required. • Pre-operative mouth rinse using 1% Hydrogen peroxide, 0.2% Povidone-iodine, 0.2% Chlorhexidine, 2% Listerine. • Rubber dam especially in aerosol generating procedures. • Avoid the use of ultrasonic, • Use hand instruments. • Avoid the use of three-way syringes if possible. • Avoid the use of high-speed hand-piece if possible. • Use high volume suction.
8	**Precautions and recommendations for orthodontic settings during the COVID-19 outbreak: A review** J Orthodont Dentofacial Orthopedics (2020), doi: https://doi.org/10.1016/j.ajodo.	Khadijah A. Turkistani, Kingdom of Saudi Arabia	• Strict infection control measures • reduce personal contact • Standard PPE including N95 or FFP2 masks • Surface Disinfection	• Emergency only • Patient online evaluation prior to treatment • Instrumentation sterilization using steam autoclaves. • Medical wastes disposal protocols • Dentists daily self-care evaluation • Mouth rinse, 0.2% chlorhexidine, prior treatment. • limit aerosol generating procedures
9	**Transmission routes of 2019-** **nCoV and controls in dental practice.** *Int J Oral Sci*. *2020;12(1)*:*9*. *doi*: *10*.*1038/s41368-020-0075-9*.	Peng X, Xu X, Li Y, Cheng L, Zhou X, Ren B. China	Prevent spread via direct transmission: coughing, droplet inhalation, sneezing, contact with mucous membranes of mouth, nose and eyes. • Transmitted directly or indirectly through high viral loads in sputum of asymptomatic patients. • Transmitted by aerosols during procedures; face-to-face contact; exposed to saliva, blood, and aerosols, on instruments	• Hand hygiene • PPE • Rubber dam • No high-speed handpieces • Four-handed Dentistry • Use handpiece with anti-retraction valve • Disinfect office handles doors
10	**Severe Acute Respiratory Syndrome (SARS) and the** **GDP. Part II: Implications for GDPs** ***British Dental Journal*** *VOL 197 NO*. *3 AUGUST 14 2004*	RWK. Li, KWC. Leung, FCS. Sun, LP Samaranayake UK	Control the patient’s gag, vomiting or cough reflex Reduction or avoidance of droplet/aerosol generation • Use Rubber Dam isolation • Use of pre-procedure mouthwash • Dilution and removal of contaminated ambient air • Disinfect air/ aerosol generated • Adoption of contact precautions	• proper patient positioning. • avoid intra-oral radiographs. • use intra-oral topical anaesthesia. • avoid high speed handpieces, scalers • good ventilation. • use of UV germicidal irradiation. • Ozone air purification. • Thorough hand hygiene • Disposable surface covering. • Surface disinfection • Disinfection of instruments and materials • Standard PPE.
11	**Infection prevention and control during health care when COVID-19 is suspected, Interim Guidance** *WHO 19 March 2020* https://www.who.int/publications-detail/infection-prevention-and-control-during-health-care-when-novel-coronavirus-(ncov)-infection-is-suspected-20200125	WHO Switzerland	• Triage, • Standard patient precaution, • Hand hygiene • PPE • Mask for patient, • Environment cleaning, disinfection, • ventilated rooms, • social distancing: -1metre apart,	
12	**Rational use of personal protective equipment for coronavirus disease 2019 (COVID-19), Interim guidance** *WHO 27 February 2020* *https://apps.who.int/iris/bitstream/handle/10665/331215/WHO-2019-nCov-IPCPPE_use-2020.1-eng.pdf*	WHO Switzerland	• Preventative measures: • Hand hygiene, Avoid touching face, • Sneeze into elbow, • Wearing masks, • Social distancing	• PPE: outline type of PPE for the different health workers, support staff, visitors and patients in a healthcare facility’s waiting room, consulting room, admin areas
13	**Physical distancing, face masks, and eye protection to prevent person-to-person transmission of SARS-CoV-2 and COVID-19: a systematic review and meta-analysis.** *www*.*thelancet*.*com* *Published online June 1*, *2020* *https://doi.org/10.1016/S0140-6736(20)31142-9*	DK Chu, EA Akl, S Duda, K Solo, S Yaacoub, HJ Schünemann Canada	*Physical distancing*: Short distance = <1m 12.8% chance infection/ transmission. With intervention = >1m 2.6% chance of inf or trans. *Face masks*: Without mask/respirators = 17.4% chance infection or transmission. With intervention: with masks or respirator = 3.1% chance of infection or transmission. *Eye protection*: Without any eye protection = 16.0% chance of infection or transmission. With intervention: with eye protection = 5.5% chance of infection/ transmission.	
14	**Oral healthcare during the COVID-19 pandemic.** *J*.*Dental Science*, *https://doi.org/10.1016/j.jds.2020.04.012*	O Lucaciu, D Tarczali, N Petrescu Romania	• Social distancing, keep appointments apart to avoid contact between patients • Hand hygiene, • Standard PPE including N95 or FFP2 masks • Remove magazines, other loose touchable objects from waiting room. • Patients to wear masks	• Minimal invasive procedures, • Pre-treatment mouth rinses, • Use of rubber dam • 4 handed dentistry. • extra-oral radiographs • Use resorbable sutures. • Limit aerosol generating procedure. • Deep disinfection of surfaces, hand-pieces, area after treatment.

KEY: PPE: Personal Protective Equipment.

**Table 3:** Outcomes of virus transmission of included studies.

	Titles	Study Details	Study Outcomes			
		Author Country	Transmission Mode	Transmission Source	Prevention of Spread of Infection	Guidelines or Recommendation for Dentists
1.	**COVID 19: Implications for dental care** J Endo. Elsevier Inc, 2020; 46(5), pp. 584–95. doi: 10.1016/j.joen.2020.03.008.	A Ather, B Patel, NB Ruparel, A Diogenes, KM Hargreaves USA	• Via respiratory droplets or by contact • Landing of droplets on inanimate objects nearby an infected individual and touched by others • Virus in saliva and faeces of the infected person • COVID-19 binds to • human angiotensin converting enzyme 2 receptors, which are highly concentrated in salivary glands	• Potential COVID-19 transmission via aerosol fomites, or faecal-oral route contributing to spread in dental office. • Asymptomatic patients act as carriers and serve as a reservoir for re-emergence of infection • Virus remain viable in aerosol and survive up to 3 days on inanimate surfaces at room temperature	• Hand hygiene • PPE • Disinfectant for hands and inanimate surfaces	• Follow standard, contact/ airborne practices, • PPE • Hand hygiene practices • Follow CDC prevention guidelines • Pre-procedural mouth wash:0.2% povidone-iodine and 0.5%-1% hydrogen peroxide mouth rinse • Use disposable devices (mirror, syringes). • Use rubber dam • Avoid stimulate gag reflex: taking radiographs • Minimize use of ultrasonic instruments, high speed hand pieces and 3-way syringes • Negative pressure rooms/ airborne infection isolation rooms recommended for positive patients.
2.	**COVID-19: impacts to dentistry and potential salivary diagnosis** Clin Oral Invest, 2020 24(4), pp. 1619–1621. doi: 10.1007/s00784-020-03248-x.2020	R Sabino-Silva, ACG Jardim, WL Siqueira Brazil & Canada	• Human–human transmission via saliva droplets when talking, coughing, sneezing (relates to human respiratory activities)	• Human-human transmission • Transmission through air suspended viral particles and contaminated blood needs to be considered. • Asymptomatic infections also a possible transmission route even before symptoms occur	• Social distancing	• Crucial for dentists to refine preventative strategies to avoid COVID-19 infection; focus on patient placement (Non-maleficence focus to avoid harm) • hand hygiene, • All PPE and • Caution in performing aerosol-generating procedures (Non-maleficence focus to avoid harm)
3	**COVID-19 outbreak: an overview on dentistry** Int J Environ Res and Pub Health, 2020 17(6), pp. 3–5. doi: 10.3390/ijerph17062094.	G Spagnuolo, D De Vito, S Rengo, M Tatullo Spain	• Transmitted through inhalation/ingestion/dire mucous contact with saliva droplets; • Virus can survive on hands, objects or surfaces that were exposed to infected saliva in the previous 9 days	• Virus can survive on hands, objects or surfaces that were exposed to infected saliva in the previous 9 days • Copper and paper allow the virus to survive for 4 to over 24 hours; Steel– 48 hours and Plastic 72 hours	• Rinsing with antiseptic mouth wash	• Dentists to avoid scheduling of any patients • Attend only to emergencies (Autonomy recognized) • Intercept potentially infected patient before they reach the operating areas (remote consultation) • Communicate to patients and dental personal to stay at home if presenting with any symptoms • Managing the operating area must similar to treating other patients infected with infectious and highly contagious diseases. • Work at distance from patients • Hand-pieces to be equipped with anti-reflux devices to avoid contamination, • Considered high risk areas: surfaces in waiting area. • Adequate periodic air exchange, and • Disinfection of all surfaces. • Air conditioning system must be sanitized often.
4	**Possible aerosol transmission of COVID 19 and special precautions in dentistry** J Zhejiang Univ: Science B, 2020 21(5), pp. 361–368. doi: 10.1631/jzus.B2010010.	Zi-yu GE, Lu-ming YANG, jia-jia XIA, Xiao-hui FU, Yan-Zhen ZHAG China	• Virus transmitted between people through close contact droplets • Airborne transmission	• Virus found on inanimate objects • 72 hours on plastic and stainless steel; 4 hours on copper; 24 hours on cardboard	• Waiting areas post cough etiquette; • Social distancing • Equipment to be disinfected between each patient • Hand hygiene • PPE • Pre-procedural mouth rinses (chlorhexidine-cetylpyridinium- chloride) • Rubber dam isolation • Removal/ filter of contaminated air • Environment surface disinfection	• Patient screening needs to become routine (medical history/ health status check/ including questions about coughing, fever, travelling or contact with COVID positive patients). • Avoid elective • procedures • Suspected or confirmed cases, reschedule till after outbreak; • If patient is positive and emergency treatment required, highest level of PPE be implemented and–negative pressure room to be utilized (min 12 air changes per hour or at least 160 L/s per patient). • Mechanical ventilation used before treating next patient.
5	**Oral Saliva and COVID-19** Oral Oncology. Elsevier, 2020 108(May), p. 104821. doi: 10.1016/j.oraloncology.2020.104821.	Maryam Baghizadeh Fini Iran	• Salivary droplets: size of the droplet determines how far and long they can travel along with the airflow.	Saliva contain saliva secreted from major and minor salivary glands as well as secretions from the nasopharynx and lung.	PPE	• Minimize treatments that produce droplets or aerosols. • Avoid elective procedures
6	**Coronavirus Disease 2019 (COVID-19): Emerging and Future Challenges for Dental and Oral Medicine** J Dent Research 2020 99(5) 481–7	L. Meng, F. Hua, Z. Bian China	Based on genetic and epidemiological research. • Started with single animal-to-human • Sustained human-to-human spread next • Currently interpersonal transmission via droplets (respiratory/ oral) and contact • Possible risk of fecal-oral transmission	• Symptomatic patients is main source • Asymptomatic patients in their incubation period can be carriers	• Effective hand hygiene • Use of disinfectant for hands and surfaces • Use of PPE	• No consensus on provision of dental services • Minimize treatments that produce droplets or aerosols. • Use of saliva ejectors. *Emergency cases*: • Rubber dams, • high volume ejectors, • Face shields and goggles essential. -High/low speed drilling with water spray.
7	**What dentists need to know about COVID-19** Oral Oncology 105, 2020, https://doi.org/10.1016/j.oraloncology.2020.104741	Maryam Baghizadeh Fini Iran	Via human interaction •coughing •sneezing •laughing; Where contaminated airborne particles are inhaled or directly brought into contact with eyes, mouth, nasal passages	• Started with a single transmission from animal • Subsequently from human spread • Symptomatic patients are main source, • Asymptomatic patients are carriers of Corona virus	• Adequate screening telephonically, • Patient assessment and care protocol (medical history, health status check), pharmaco-logical treatment 1^st^ • next physical dental treatment, using dental guidelines, • Treat emergencies • adequate hand washing protocols	• Avoid touching unnecessary areas (dentures, bite blocks) • Disinfect work every time, • avoid procedures causing coughing or gag reflexes Clinical tips: • Do tooth extractions in a supine position (prevents from operating in respiratory tract). • During CRD or RPDs try-in, stop touching items in dental workplace after contacting saliva. • Prosthodontic material (e.g., RPD, impressions) to be disinfected by intermediate level disinfectant. • Salivary suction carefully done, use topical anaesthesia when choosing size/ modifying impression trays to prevent gag reflex.
8	**Rational use of personal protective equipment for coronavirus disease 2019 (COVID-19), Interim guidance** *WHO 27 February 2020* https://apps.who.int/iris/bitstream/handle/10665/331215/WHO-2019-nCov-IPCPPE_use-2020.1-eng.pd*f*	WHO Switzerland	• Virus transmitted between people via close contact droplets • Airborne transmission	• Virus can be found on inanimate objects • 72 hours on plastic and stainless steel • 4 hours on copper • Up to 24 hours on cardboard		• WHO recommendations for the rational use of PPE in health care and home care setting, • Handling of cargo, as well as • Assesses the current disruption of the global supply chain and • consideration for decision making during severe shortages of PPE (Beneficence addressed)

KEY: PPE: Personal Protective Equipment; CRD: Complete removable denture; RPD: Removable partial denture.

**Table 4:** Outcomes of Ethical principles of included studies.

	Titles	Study Details		
		Author Country	Ethical Principles	Guidelines or Recommendation for Dentists
1	**Guidance on continuing to practice ethically during COVID 19** ADA 26 March 2020	ADA, USA	**Autonomy**: Duty to respect the patient’s rights to self-determination and confidentiality, safeguard confidentiality **Non- maleficence**: Duty to refrain from harming the patient **Beneficence**: Promote the welfare of patients **Justice**: Fairness in health-related decisions **Veracit**y: Duty to communicate truthfully	• Guidelines for dentists to maintain high ethical standards during COVID-19 • Compromise some core principles for the greater good • Individual versus Public rights
2	**Ethical moments: Ethical practice during the COVID-19 pandemic** JADA 2020 151 (5): 377–78	Cohen USA	Autonomy, non- maleficence, ethical principles	• Principles of Ethics and • Code of Professional Conduct
3	**COVID 19: Balancing personal risk and professional duty** BMJ 369: m1606	Harkin DW UK	**Hippocratic Oath**: “Act for patients’ benefit and do no harm” Patients welfare, autonomy Social justice, public trust and professional codes	• Ensure medical professionalism, • Balancing personal risk and professional duty
4	**COVID-19: Where is the national ethical guidance?** BMC Medical Ethics 2020 21 (1): 32	Huxtable R UK	• Fairness distribution, • cash out principle • more localised decision making	• NATIONAL guide is necessary for decision-making • Resource allocation guidance
5	**Coronavirus disease (COVID-19) outbreak: Rights, Roles and Responsibilities of health workers** WHO /2019-nCov/HCW. advice/2020.2	WHO Switzerland	• Health worker rights, roles and responsibility	• Highlights rights, responsibilities of health workers, • Education suited for a pandemic • measures needed to protect occupational safety and health.

The extracted data was interpreted in a manner where it addressed the objectives of the review and the findings are synthesised narratively. The data extracted were not from primary studies as no SARS-CoV-2 or COVID-19 related research was completed in the dental setting when this review was conducted. The information retrieved were largely from country specific oral health groups, health policymakers and dental organizations to provide guidance to dental practitioners during such an unprecedented pandemic. The characteristics of the included studies are summarised in Tables 2, 3 and 4 and are reported under the specific focus areas or themes for this scoping review. The studies that were excluded comprised of editorials with little or no information that could be used, few were unrelated to the areas of review interest, some were in a foreign language and it was not possible to retrieve the others.

### Infection control within the dental setting

Only one infection control article reported on a cross-sectional survey where a questionnaire was sent to practitioners [18]. Gauging from the results of searches, the other articles were either reports, recommendations or reviews, including a systematic review, or guidelines developed during another infection outbreak, as these could be followed during this unprecedented COVID 19 pandemic [1, 6, 18–29]. But no papers focused on primary research or interventions or were control studies nor on the effectiveness of infection control measures used in dental practice to curb the transmission of COVID-19. However, even though the nature of the research obtained were of inferior quality, it could serve as a guide for practitioners and dental hospitals in the absence of COVID 19 specific research as it was found that the infection control measures reported are similar to that adopted against the spread of MERS and SARS [25].

In Dentistry, infection control measures are evidence-based and standard operating procedures are generally strictly followed, but it is the first time in decades that the dental profession has been confronted with a pandemic. What the current global situation has permitted the profession to do is relook at the existing infection control measures and identify studies to be conducted on how these may be optimized to prevent transmission of the SARS-CoV-2 [19–24, 29]. More importantly, due to the nature of COVID 19, these standard operating procedures may have to be modified according to the type of clinical work dentists undertake, such as, aerosol or non-aerosol generating techniques and within the related clinical dental settings (even specialties) after gauging all the different areas that are impacted.

More specifically, and according to the literature obtained, infection control measures mostly referred to were: personal protective equipment (PPE) such as masks, gloves, eye-wear and gowns; hand hygiene; pre-procedural mouth rinses; four-handed dental procedures; avoidance of aerosol generating procedures; use of extra-oral radiography and disinfection of all surfaces and other outside clinical areas commonly touched by staff and patients [1, 19–24, 26–29]. But from the evidence, there are very many variations to each aspect mentioned here, such as, what type of mouth-rinse is referred to or the extent of PPE recommended which would depend on the type of aerosol versus non-aerosol procedures clinicians are engaged in (Table 2) [1, 6, 17–28]. Disinfecting procedures (when, how, frequency, areas, equipment and who) and adequate ventilation of consulting and treatment rooms are also mentioned (Table 2) [1, 19, 22]. In addition, many preventive measures were recommended including online patient assessments, screening and triage of patients for emergency treatments, but without providing any details of these (Table 2) [20, 22]. Preventive measures against infection transmission were referred to in many of the articles such as social distancing, waiting room changes and patient preparations, such as pre-treatment oral rinses (Table 2) [1, 19, 23].

### Transmission within the dental setting

The seven unstructured transmission reviews principally discussed several measures on how to protect staff and patients from becoming infected with SARS-CoV-2 or COVID-19 [1, 6, 9, 20, 30–33].

The discussion in the included literature at the time of the searches for this scoping review, mostly relates to spread via social interaction such as where the virus attaches, how it attaches on the different surfaces within the clinical setting, the types of actions resulting in this (sneezing, coughing, talking) and how dental practices need to include extra precautionary measures to mitigate or even prevent this [10, 30, 32–33]. It has been reported that the virus remains on hands, objects and surfaces that has been exposed to infected saliva in the previous 9 days (Table 3) [21, 31]. The survival periods in hours of the virus on the different types of surfaces or materials such as paper, plastic, steel and cardboard has also been reported (Table 3) [10, 27, 31, 33]. This is of particular importance to staff and use of materials in the dental setting, including test books and daily worksheets. It is further advised to therefore avoid touching surfaces as much as possible or to delay touching these surfaces where required.

There were several recommendations to practitioners with regards to prevention of infection transmission, different treatment protocols for patient treatment (aerosol versus non-aerosol procedures) and awareness about handling dental work (extra disinfecting of all impressions and orthodontic and wax work), the dental environment and dentist self-care (Table 3) [1, 6, 10, 28–33]. The separation between the types of clinical procedures is necessary as it has been reported that the virus survives in aerosols for up to 3 days at room temperature and more importantly, asymptomatic patients may be carriers (Table 3) [21]. Therefore, recommendations also include special pre-procedural oral mouthwashes for all dental disciplines, use of rubber dam to isolate the working area in aerosol generating clinics, limited handling of dental work, use of disposable mirrors, high volume ejectors and avoidance of procedures where high-speed equipment creating aerosols are required (Table 3) [10, 30, 32]. Discipline specific recommendations were also shared, such as disinfection guidelines for dentures, wax record blocks and impressions in Prosthetic dentistry which was of particular interest to the researchers [1, 21, 30].

### Ethical principles as it relates to the dental professional during a pandemic

Dentists are guided by ethical principles, and at the start of the COVID-19 pandemic the recommendation to cease all dental procedures immediately and refuse patients any kind of elective dental treatment may be regarded as acting against these very values they supposedly commit to [2, 7, 14]. But these guidelines during such an unprecedented pandemic served to focus dental practitioners to save lives of individuals due to the absence or minimal scientific evidence present related to COVID-19 [34]. It has been recognized that country specific national guidelines be developed as a directive to dental practitioners regarding patient treatment and clinical practice decision-making during the COVID-19 pandemic, whilst any deviations from the very ethical principles required, be endured by all [34, 35]. For example, denying patients elective dental procedures in order to contain the spread of the virus is ethically acceptable as individual interests may be disregarded to ensure the safety of the public during a pandemic [36].

More importantly, it is advised (and be seen as compulsory ethical actions) to inform patients presenting at the dental practice about the risks of the viral infection to them, their families and the community at large [37]. Therefore, an easing on ethical principles such as patient confidentiality is accepted (where patients COVID-19 status can be revealed) though the expectation is that practitioners will still be maintaining high standards of care [35]. However, non-conforming behaviours do not imply that any unprofessional behaviour would be tolerated nor any deviations from the code of professional conduct [35]. More importantly, it has been stated that the health and welfare of frontline healthcare workers, their rights and responsibilities should never be compromised whilst treating patients, whose well-being is dependent on them due to the respect, confidence and trust all communities place on them, especially when dealing with emergencies that may arise [14, 34].

In summary, disinfection measures discussed includes how patients and staff and waiting rooms and clinical areas are impacted; and what now needs to change from normal protocols and for the different types of procedures (emergency, aerosol and non-aerosol generating procedures and for the different specialties) and how each of these may vary, especially within a hospital setting. Moreover, this review allowed a refocus on normal everyday disinfection procedures with one goal in mind, and that is to stop the spread of COVID-19 whilst maintaining ethical principles and act with the patients’ and staff best interest at heart [1, 6, 9, 17–35].

## Discussion

Dental practitioners, like all other healthcare workers, are guided by taking an oath to serve and protect their patients, especially saving their lives [9, 36]. This is the mind-set required during a pandemic such as COVID-19. Dentists are obliged under these ethical principles to safeguard the confidentiality of patient records, but during the pandemic, they will have to relinquish this obligation if contact tracing and/or reporting of positively diagnosed patients become necessary to reduce the spread of COVID-19 [34]. During a pandemic, patient autonomy, confidentiality, consent and individual benefits must be overlooked against ensuring public safety and risk reduction on all fronts, such as requests for elective treatment. Whilst saying this, it is most important to acknowledge that trust and honesty should not be compromised during a pandemic and that a balance between personal risk and professional duty must be observed.

Although most of the public attention is focusing on the direct causes, transmission and control measures of SARS-CoV-2, possible health consequences resulting from people’s natural fears of being infected should not be overlooked. Understanding the current situation regarding dental practice and the limitations it had to endure, it must be acknowledged that it would be helpful in terms of predicting future dental needs. Patients’ fear of these routes of spread of COVID-19 has limited their visits to the dentists for extreme emergency procedures only, as expected. But based on the results of this review, we have reasons to speculate that people’s requirements for dental services might grow explosively whilst still in this COVID-19 period. The strengths of administrative and health departments of the government are suggested to be coordinated to implement comprehensive prevention and control measures for future dental care. In addition, dentists must avail themselves to patients who require dental emergency management, irrespective of whether they have been positively diagnosed with COVID-19 or not [6, 7]. Therefore, dental staff will have to be updated regarding the infection control and transmission of infection measures and be encouraged to practice stricter and more effective disinfection measures, even though it would take more time and effort to complete [1].

Standard infection control precautions which include hand hygiene where evidence suggests that 20–40 seconds of washing with soap and water is enough to destroy COVID-19 needs to be re-emphasized [1, 3, 5, 9, 10]. Hand hygiene not only includes washing with soap and water, but also the use of a hand sanitizer containing 70% alcohol, and these are considered a crucial practice to control the spread of COVID-19 [1, 30–32]. Comprehensive mouth cleaning routines for patients should also be enforced to include brushing, flossing, mouth rinsing (using special pre-oral mouth washes) and soaking the tooth brush in bleach for 30 minutes [30–32]. The use of pre-procedural mouth rinses must especially be the routine in the dental setting as it prevents the spread of the virus by reducing the number of microorganisms that a patient may release (from their saliva) which is created during splatter and within aerosols plus standard infection control measures when dealing with all patients during a pandemic. Additionally, the PPE that includes, masks, gloves, gowns, goggles or face shields should be worn by dental personnel to protect them from coming into contact with blood, saliva and droplets, thus preventing cross infection for all aerosol and non-aerosol generating procedures [30–33].

In addition, and more specifically, many variables could alter the microbiological composition of the oral environment, such as prosthodontic frameworks, orthodontic appliances, such as the influence of lingual bracket position on microbial and even periodontal parameters in vivo, or paediatric treatments or dental implants [38–42]. Therefore, in the future the role of these variables and how these are spread should also be considered, taking into careful account their potential effects [43]. Some of these serious modifications include re-evaluating and renaming procedures according to the level of infectiousness and the appropriate protection required accordingly [43]. Moreover, the modifications required within existing operating measures and for the different dental disciplines and specialities will have a serious impact on patients, their future dental needs and requirements and maintenance, including their quality of life [41–45].

Since the outbreak of COVID-19, most countries have reacted to and focused on preventing the transmission of the virus following public health measures, thus the emphasis on ensuring effective transmission-based precautions. It has been established and confirmed that the transmission routes are via contact (human and surface contacts), droplet and airborne spread and many of these were described in the articles reviewed and are recorded on the Tables [1, 6–10, 19–33]. It is well known that many dental procedures regularly produce aerosols and droplets which normally may be contaminated with bacteria, viruses and blood and may easily spread to the dental personnel and other patients within these settings [7–10]. In addition, the contact between different surfaces that naturally occur in the dental setting may further contribute to the spread of any harmful substances, including COVID-19 from unsuspected or asymptomatic patients or dental personnel.

Within the confines of the current global pandemic, some of the ethical guidelines will have to be renounced as it is a matter of public versus individual health risks, as stated above. There should be mutual respect for all involved, and prevention of harm refers to that of the collective and not just the individual attending a dental practice. It is not unusual that during a pandemic, the required equipment will not be available or dentists will have to overlook particular protocols as long as it is for the benefit of patients, but at the same time they will still have to observe these ethical principles. Thus, dental emergency services must be reframed ethically as practitioners have a greater responsibility towards the public at large. They may change practices and operating procedures according to the need of the community, resources available and changing practice related to staff [14, 34–37]. All these deviations from the norm in ethical practices, directly speaks to an ethical framework that is forced to change during a pandemic.

The strengths of this review include identification of clinical areas (physical space and treatment procedures) normally overlooked and the specific attention required to prevent transmission of COVID-19. Moreover, it highlights the need for a change or modification in normal everyday procedures, such as disinfection, infection control, prevention of infection transmission and the set ethical practices and including conducting more focused research as it relates to these areas mentioned. One example would be the many different mouth rinses and whether the use of these would be viable to limit the spread of SARS-CoV-2. In addition, researchers are aware of the dearth of clinical research due to the nature of the pandemic, but also of the barrage of information related to the clinical setting that surfaced in recent times as clinicians and academics were searching for answers on how to manage and function during this unprecedented period [41–45]. Thus the approach and focus adopted for this review also speaks to the weakness of the available literature for the scoping review, which is further alluded to under the limitations as well.

### Limitations

One major limitation of the review is the assessment of the quality of the included research reports with subsequent limited appraisal of included articles [46]. This is attributed to the fact that these articles were mostly from the base of the evidence pyramid, which is normally regarded as poor evidence. For this scoping review, a critical appraisal using a standardized tool was not completed following the guidelines set for this type of review [15–16]. Thus, none of the included articles may be considered as good evidence and or even translatable into practice based on the evidence pyramid.

Moreover, the rate at which researchers were completing reviews and opinion papers during this pandemic spiked, as most of them engaged with such types of research as it was not possible to conduct primary research. Thus, for this review, many of the published articles post this data collection stage, would not be included here. In addition, the outcomes and methods were inconsistent throughout all included studies and as stated above, no primary studies were conducted during the pandemic, thus no strong conclusions could be drawn. Whilst saying this and critically viewing the obtained articles or ‘evidence’ for this review, we must be cognizant of the guidelines that clinical practitioners sorely and urgently needed during this COVID-19 pandemic. Thus, it does not mean that the ‘evidence’ obtained for this scoping review, even though considered of poorer quality, may not be considered or used as a guide during this unprecedented COVID-19 pandemic.

Other limitations noted for this review, is the focus on the basic general dental procedures and not those highlighting differences within the dental disciplines, such as orthodontics, paediatric dentistry or geriatric clinics [39–42]. With the new information published during the COVID 19 pandemic, researchers were also addressing alternatives to basic operations in practice and related to the teachings at institutions [47–49]. These include available alternatives such as tele-dentistry instead of face-to-face consultations which should also have been emphasized, though it is currently not feasible due to the large numbers of underprivileged patients seen at the practice setting and country where this review was completed. It is acknowledged though as a promising modification to improve access and communication during such unprecedented periods and even for non-crisis settings to minimize the risk of cross-infections and/ or transmission of infections [47, 48]. Tele consultation, tele-diagnosis, tele-triage, and tele-monitoring are subunits of tele-dentistry and must be included in the curriculum; students trained to practice these options and practiced across the different levels and disciplines [47, 48]. Another limitation that was not discussed but needs to be emphasized is how a digital workflow using computer-aided design/computer-aided manufacturing (CAD/ CAM) technology may be used during this COVID 19 period to limit infection risk for prosthetic and prosthodontic treatments [49]. This aspect will form part of other research related to digital workflow specifically, thus it was excluded.

### Recommendations

Within the confines of the research question and the objectives of this review, the focus on future research should include:

- Clear guidelines as to what constitutes a dental emergency,
- More clarity on what triaging in Dentistry entails (online and face-to-face),
- The length the corona virus is viable on different surfaces within a dental practice,
- How long it takes to eradicate a corona virus following disinfection using standard infection control measures of the different surfaces in a dental setting,
- Development of a new ethical framework appropriate for a pandemic,
- Ethically acceptable clinical dental research with corona virus infected patients and
- The role of a digital workflow (partial or complete) and its utilization during a pandemic to prevent cross infections [49].

## Conclusion

Infection control measures and transmission of the SARS-CoV-2, including the modes and source and prevention measures were discussed in detail, and implementation of these were emphasized. But it is clear that no quality evidence is available that can support the positions recommended or stated to prohibit transmission of infections, but these may be used to mitigate patients becoming infected. A different ethical framework is required during a pandemic which must be informed by evidence.

## Supporting information

S1 File Preferred reporting items for systematic reviews and meta-analyses extension for scoping reviews (PRISMA-ScR) checklist. (DOCX)

S2 File (DOCX)
