# Supplementary material for: COVID-19 and its impact in the dental setting: A scoping review
Source: PLoS One. 2020 Dec 18;15(12):e0244352. doi: 10.1371/journal.pone.0244352 (PMC7748282; doi:10.1371/journal.pone.0244352)
Supplement: S2 File — (DOCX) [file pone.0244352.s002.docx]

A: **Search Terms per Databases**

Infection Control Section:

PUBMED: *Covid 19, Coronavirus, infection control, dental, dentistry,*

ELSEVIER: *Covid 19, Coronavirus, infection control, dental, dentistry,*

WILEY: Coronavirus,+covid+19,+infection+control,+dental,+dentistry

SCIENCEDIRECT: *Covid 19, Coronavirus, infection control, dental, dentistry,*

Transmission of Infections section:

PUBMED: COVID 19 and Dentistry and Transmission of infections

Scopus: COVID 19 transmission and Dentistry

Science Direct: COVID 19 transmission and Dentistry

WHO website: COVID 19 transmission and Dentistry

Ethics section:

PUBMED: COVID 19 and Ethics

WILEY: COVID 19 and dental ethics

Science Direct: COVID 19 and dental ethics

WHO website: COVID 19 and dental ethics

*EXAMPLE of a completed search strategy*:

(COVID-19 OR corona virus OR SARS-CoV-2) AND (dental clinics OR dental hospital OR dental institutions OR dental teaching clinic) AND (infection control OR disinfection) AND (literature reviews OR reviews OR observational OR clinical trials OR randomized controlled trials OR qualitative research OR systematic reviews) AND (2015/01/01-2020/05/30.

B: **SCALES used for the Scoping Review**

*Years*: 2015 to 2020 (we completed the searches before June 2020)

*Study designs*: Included all different types of study designs (primary and secondary)/ reports/ Recommendations/ Guidelines

*Setting*: dental setting, Oral health sectors

*Language*: Only English

*Databases*: Included scientific publication databases but also Health Group websites, Dental Organizations and Dental Scientific Groups’ sites.
